# Galacto-oligosaccharides improve barrier function and relieve colonic inflammation via modulating mucosa-associated microbiota composition in lipopolysaccharides-challenged piglets

**DOI:** 10.1186/s40104-021-00612-z

**Published:** 2021-08-11

**Authors:** Ren Gao, Shiyi Tian, Jing Wang, Weiyun Zhu

**Affiliations:** National Center for International Research on Animal Gut Nutrition, Jiangsu Key Laboratory of Gastrointestinal Nutrition and Animal Health, National Experimental Teaching Demonstration Center of Animal Science, Laboratory of Gastrointestinal Microbiology, College of Animal Science and Technology, Nanjing Agricultural University, Nanjing, 210095 China

**Keywords:** Barrier function, Galacto-oligosaccharides, Intestinal microbiota, Lipopolysaccharides, Suckling piglets

## Abstract

**Background:**

Galacto-oligosaccharides (GOS) have been shown to modulate the intestinal microbiota of suckling piglets to exert beneficial effects on intestinal function. However, the modulation of intestinal microbiota and intestinal function by GOS in intestinal inflammation injury models has rarely been reported. In this study, we investigated the effects of GOS on the colonic mucosal microbiota composition, barrier function and inflammatory response of lipopolysaccharides (LPS)-challenged suckling piglets.

**Methods:**

A total of 18 newborn suckling piglets were divided into three groups, the CON group, the LPS-CON group and the LPS-GOS group. Piglets in the LPS-GOS group were orally fed with 1 g/kg body weight of GOS solution every day. On the d 14, piglets in the LPS-CON and LPS-GOS group were challenged intraperitoneally with LPS solution. All piglets were slaughtered 2 h after intraperitoneal injection and sampled.

**Results:**

We found that the colonic mucosa of LPS-challenged piglets was significantly injured and shedding, while the colonic mucosa of the LPS-GOS group piglets maintained its structure. Moreover, GOS significantly reduced the concentration of malondialdehyde (MDA) and the activity of reactive oxygen species (ROS) in the LPS-challenged suckling piglets, and significantly increased the activity of total antioxidant capacity (T-AOC). GOS significantly increased the relative abundance of *norank_f__Muribaculaceae* and *Romboutsia*, and significantly decreased the relative abundance of *Alloprevotella*, *Campylobacter* and *Helicobacter* in the colonic mucosa of LPS-challenged suckling piglets. In addition, GOS increased the concentrations of acetate, butyrate and total short chain fatty acids (SCFAs) in the colonic digesta of LPS-challenged suckling piglets. GOS significantly reduced the concentrations of interleukin 1β (IL-1β), interleukin 6 (IL-6), tumor necrosis factor-α (TNF-α) and cluster of differentiation 14 (CD14), and the relative mRNA expression of Toll-like receptor 4 (*TLR4*) and myeloid differentiation primary response 88 (*MyD88*) in the LPS-challenged suckling piglets. In addition, GOS significantly reduced the relative mRNA expression of mucin2 (*MUC2*), and significantly increased the protein expression of Claudin-1 and zonula occluden-1 (ZO-1) in LPS-challenged suckling piglets.

**Conclusions:**

These results suggested that GOS can modulate the colonic mucosa-associated microbiota composition and improve the intestinal function of LPS-challenged suckling piglets.

**Supplementary Information:**

The online version contains supplementary material available at 10.1186/s40104-021-00612-z.

## Introduction

The intestinal microbiota of pigs play a key role in regulating host digestion, absorption, intestinal barrier function, and the maturation of the immune system [1]. Studies have shown that the intestinal microbiota of pigs are established early in life, and the changes in the intestinal microbiota early in life can have permanent metabolic consequences for the host. Additionally, the establishment of early-life intestinal microbiota is susceptible to nutritional conditions [2, 3]. It is suggested that the early nutrition is a primitive “driving force”, which modulates the colonization of the intestinal microbiota and the development of the intestinal immune system. In the pig industry, intestinal epithelial cells of young animals are vulnerable to inflammation and infection, which negatively affect the health of animals [4]. Therefore, obtaining a stable and healthy intestinal microbial community status through nutritional intervention in early life is of great significance for the subsequent growth and health of animals.

Lipopolysaccharides (LPS) are composed of lipids and polysaccharides in the outermost layer of the cell wall of gram-negative bacteria and considered as a strong inflammatory stimulant [5]. LPS activate Nuclear factor kappa B (NF-κB) by inducing a signaling cascade that leads to the secrete of a series of inflammatory factors, and ultimately induce the colonic inflammatory response in piglets [6, 7]. LPS-induced intestinal inflammation is a common animal inflammatory injury model [8]. As research on the intestinal microbiota continues to deepen, researchers have found a close relationship between intestinal inflammatory diseases as well as intestinal health and intestinal microbiota in piglets [9]. The increasing demand for antibiotic-free animal production has led many investigators to pay more attention to how to modulate the intestinal microbiota of suckling piglets with nutritional strategies to achieve the purpose of maintaining the intestine healthy. Galacto-oligosaccharides (GOS) are a type of functional oligosaccharide with natural properties presented abundantly in human breast milk and in trace amounts in porcine and other animal breast milk [10, 11]. As a common prebiotic, GOS are not digested and hydrolyzed in the small intestine because of their structural characteristics, but enter the hindgut, thereby are fermented and utilized by microorganisms to promote the proliferation of beneficial bacteria and contribute to the stability of intestinal microbiota. Therefore, GOS have received more attention in recent years [12]. Currently, GOS are mainly produced by hydrolysis of lactose through the catalytic activity of glycoside hydrolases and have been rarely applied in suckling piglet feed additives [13]. Some studies have reported the benefits of GOS supplementation in suckling piglets. For example, the supplementation of GOS in milk replacer can promote the balance of intestinal microbiota development, improve intestinal morphological structure and stimulate intestinal defense mechanisms in neonatal piglets [14]. Tian et al. reported that oral administration of GOS solution in suckling piglets could promote jejunal functional development and had a positive effect on growth performance [15]. Our previous studies have shown that the early GOS intervention can modulate colonic microbiota in suckling piglets and improve biological functions [16]. Therefore, the effects of GOS in the intestinal inflammation model of suckling piglets are worthy of further exploration, which will provide a good theoretical support for the development and utilization of GOS as a feed additive.

Here, we hypothesize that GOS could modulate the intestinal microbiota of suckling piglets, alleviate inflammatory injured and maintain intestinal barrier function. In order to further understand and highlight the beneficial effects of GOS on the intestinal microbiota and intestinal function of piglets, we used LPS challenge suckling piglets to construct an intestinal inflammation model, and investigated the effects of GOS on colonic microbiota, inflammatory response and colonic barrier function in LPS challenged suckling piglets.

## Materials and methods

### Experimental design

The experimental protocol was approved by the Animal Care and Use Committee of Nanjing Agricultural University, and all animal care procedures were performed in accordance with Chinese Guidelines for Animal Welfare. In this study, 2 litters of Duroc × Landrace × Large White newborn suckling piglets with an average birth weight of 1.57 ± 0.04 kg were selected, with 9 pigs per litter. The piglets were fostered by their own mothers. Then, each litter of piglets was divided into CON group, LPS-CON group and LPS-GOS group, with 3 pigs in each group. All piglets were fed with sow and were free to obtain sow milk and drinking water during the experiment. Starting from the first day after birth, piglets in the LPS-GOS group were orally gavaged with 1 g/kg body weight (BW) of GOS in the form of solution daily, and the CON group and the LPS-CON group were gavaged with equal amounts of sterile saline. The solution was carefully injected into the mouth of the piglets with a sterile medical plastic syringe. After continuous gavage for 13 d, the piglets in the LPS-CON group and the LPS-GOS group were intraperitoneally injected with 80 μg/kg BW of LPS solution on d 14, and the piglets in the CON group were injected with an equal volume of sterile saline. 90% pure food-grade GOS was used in this experiment, which was purchased from Quantum Hi-Tech Biological Co., Ltd. (Guangdong province, China). The GOS product is synthesized artificially and contains oligosaccharides with a degree of polymerization (DP) of 2–8, approximately 90% (w/w) GOS, 8.5% (w/w) lactose, and 1.5% (w/w) glucose on a dry matter basis. Lipopolysaccharides (LPS, from *E. coli* O55:B5) were purchased from Sigma-Aldrich (St Louis, USA).

### Sample collection

2 h after intraperitoneal injection of LPS solution or sterile saline on d 14, piglets were euthanized by injection of excessive pentobarbital. The colon was separated after dissection, and the middle colonic digesta was collected and placed in a sterile cryopreservation tube and stored in liquid nitrogen at − 80 °C until analysis. Then the colon tissue was rinsed with cold sterile phosphate buffer saline (PBS). After PBS cleaning, the colonic mucosa was scraped with sterile slides and stored in liquid nitrogen at − 80 °C until analysis.

### Histomorphological analysis of colonic epithelium

The mid-colonic tissue samples were fixed with 4% paraformaldehyde solution and then processed with paraffin embedding technique. The paraffin-embedded samples were cut into approximately 6 μm thick sections and finally stained with hematoxylin and eosin to obtain colonic morphology [17]. Crypt depth and mucosal thickness were assessed in 15 well-oriented crypts and adjacent mucosa using an image analysis software (Image-Pro Plus 6.0, Media Cybernetics, USA).

### Colonic mucosal antioxidant property determination

0.1 g colonic mucosa tissue with 1,000 μL of pre-cooled PBS was homogenized to extract total protein. The crushed tissue was centrifuged at 10,000 r/min for 15 min at 4 °C, and then the supernatant was collected for further analysis. The protein concentration was determined by bicinchoninic acid (BCA) assay method with the protein detection kit (Biosharp life science, Hefei, China). Finally, the concentration of malondialdehyde (MDA), the activities of reactive oxygen species (ROS), glutathione peroxidase (GSH-Px), superoxide dismutase (SOD) and total antioxidant capacity (T-AOC) in the colonic mucosa were determined using kits (Nanjing Jiancheng Bioengineering Institute, China) and following their instructions.

### 16S rRNA analysis of colonic mucosa-associated microbiota

0.3 g colonic mucosa was used to extract the total bacterial DNA, and PowerSoil DNA Isolation kit (MoBio Laboratories, San Diego, U.S.) was used to isolate total DNA according to its operating instructions. The concentration and quality of DNA were determined using Nano-Drop 1000 spectrophotometer (Thermo Fisher Scientific, Waltham, Massachusetts, USA), and the extracted DNA was stored at − 80 °C until the next analysis. The forward primer 338F (ACTCCTACGGGAGGCAGCAG) and reverse primer 806R (GGACTACHVGGGTWTCTAAT) were used to amplify the V3-V4 region of the bacterial 16S rRNA gene. DNA was amplified using ABI GeneAmp 9700 PCR Thermocycle Instrument (Applied Biosystems, Inc., Carlsbad, USA) with specific reaction parameters as follows: first pre-deformation stage (3 min at 95 °C), then 30 s at 95 °C; 30 s at 55 °C; 45 s at 72 °C for 27 cycles, and finally 10 min at 72 °C. Next, the PCR products were purified by 2% agarose gel electrophoresis, and the PCR products were cut and recovered using the AxyPrep DNA Gel Recovery Kit (Axygen, Union City, USA). According to the preliminary quantitative results of electrophoresis, the PCR products were quantified by QuantiFluor-ST Handheld Fluorometer with UV/Blue Channels (Promega, Madison, Wisconsin, USA). Finally, the PCR product was sequenced on Miseq (Illumina Inc., San Diego, USA) after concentration normalization. The 16S rRNA raw reads in this study were uploaded to GenBank in NCBI and the accession number is SRP297862.

### Bioinformatics analysis

The PE reads obtained by Miseq sequencing were spliced according to the overlap relationship, and the sequence quality was controlled and filtered at the same time. Operational taxonomic units (OTUs) were clustered with 97% similarity cut-off using Usearch (version 7.0.1090, http://www.drive5.com/uparse/). Each 16S rRNA gene sequence was taxonomically analyzed against the Silva 16S rRNA database (Release132 http://www.arb-silva.de), using the RDP Classifier (version 2.2 http://sourceforge.net/projects/rdp-classifier/) Bayesian algorithm with a confidence threshold of 70%. Alpha diversity of colonic mucosal microbiota was analyzed with Mothur (version 1.30.2, https://www.mothur.org/wiki/Download_mothur), including Shannon, Simpson, Ace and Chao indexes. Principal coordinate analysis (PCoA) was performed based on bray-curtis distance, and then the analysis of ANOSIM was conducted based on bray-curtis distance to assess significant differences among samples using Qiime (version 1.9.1, http://qiime.org/install/index.html).

### Measurement of pH value and microbial metabolites in colonic digesta

During the sampling process, the middle colonic digesta was collected with sterilized centrifuge tubes, and the pH value was determined immediately with a pH meter (PB-10, Sartorius Group, Gottingen, Germany). According to the description of Shi et al., a gas chromatography method was used to determine the concentrations of acetate, propionate, isobutyrate, butyrate, isovalerate, valerate and total short chain fatty acids in colonic digesta [18]. The concentration of lactic acid in colonic digesta was determined by using the reagent kit (Nanjing Jiancheng Technology Co., Ltd., Nanjing, China) according to its operating instructions.

### Quantitative real-time PCR

The total RNA was extracted from 0.2 g colonic mucosa with TRIzol reagent (Takara Bio, Japan) according to the operating instructions. A Nano-Drop 1000 spectrophotometer (Thermo Fisher Scientific, Waltham, Massachusetts, USA) was used to determine the concentration and quality of RNA. The optical density value (260/280 nm) between 1.8 and 2.0 indicates that the RNA is pure and can be used for subsequent analysis.

After concentration normalization, RNA was reversely transcribed into cDNA using HiScript * III RT SuperMix for qPCR reagent kit (Vazyme Biotech, Nanjing, China) and stored at − 80 °C. The reverse transcription reaction system was 4 μL 4 × gDNA wiper Mix, 2 μL RNA and 10 μL nuclease-free double distilled water, and the reaction was performed at 42 °C for 2 min on a cycler thermal cycier (A300 Fast Thermal Cycler, LongGene, Hangzhou, China). After the reaction was completed, the sample tube was removed and 4 μL 5 × HiScript III qRT SuperMix was added into each well for another reaction, and the reaction program was 37 °C for 15 min, followed by 85 °C for 5 s.

The target genes were detected by fluorescence quantitative PCR using ChamQ SYBR qPCR Master Mix reagent kit (Vazyme Biotech, Nanjing, China) on ABI StepOnePlus real-time fluorescence quantitative PCR instrument (Applied Biosystems, Inc., Carlsbad, USA). The PCR reaction system is 20 μL including 10 μL 2 × ChamQ SYBR qPCR Master Mix, 0.4 μL forward primer, 0.4 μL reverse primer, 0.4 μL 50 × ROX Reference Dye 1, 2 μL cDNA and 6.8 μL double distilled water. The PCR process was 95 °C for 30 s, followed by 40 cycles at 95 °C for 10 s, 60 °C for 30 s, and finally 95 °C for 15 s, 60 °C for 60 s, 95 °C for 15 s. With 3 replicates for each sample. Primers for this experiment were synthesized in Invitrogen Life Technologies (Shanghai, China) and their sequences are shown in Additional file 1: Table S1. Glyceraldehyde-3-phosphate dehydrogenase (GAPDH) was used as the housekeeping gene. Finally, the relative expression of the target gene was calculated by the 2^-ΔΔCt^ method [19].

### Measurement of mucosal cytokine and cluster of differentiation 14 (CD14)

0.1 g colonic mucosa tissue with 1,000 μL of pre-cooled PBS was homogenized to extract total protein. The crushed tissue was centrifuged at 10,000 r/min for 15 min at 4 °C, and then the supernatant was collected for further analysis. The protein concentration was determined by BCA assay method with the protein detection kit (Biosharp life science, Hefei, China). Finally, the concentration of interleukin 1β (IL-1β), interleukin 6 (IL-6), interleukin 8 (IL-8), interleukin 10 (IL-10), tumor necrosis factor-α (TNF-α), transforming growth factor-β (TGF-β) and CD14 in the colonic mucosa was determined using the Enzyme Linked Immunosorbent Assay (ELISA) kit (R&D Systems Inc., MN, USA) according to its operating instructions.

### Western blot analysis

0.1 g of colonic mucosal tissue in 500 μL RIPA lysis buffer with 1% protease inhibitors (FUTURE SCIENTIFIC INNOVATION, Nanjing, China) was homogenized at 4 °C. The pulverized tissue was centrifuged at 12,000 *g* for 15 min at 4 °C, and the supernatant was collected for western blot analysis. For normalization, the protein concentration was determined with the BCA protein assay kit (Biosharp life science, Hefei, China). After normalization, 5 × loading buffer in a ratio of 1:4 was added and the proteins were boiled for 5 min to denature.

The proteins were separated by 12% dodecyl sulfate–polyacrylamide gel electrophoresis (SDS-PAGE) after cooling. The 10 × running buffer is prepared by dissolving 30 g Tris base, 144 g glycine and 10 g SDS in double-distilled water to 1,000 mL. When used, it is diluted to 1 × running buffer. The electrophoresis process was 80 V constant pressure for 30 min, and then changed to 120 V constant pressure, until the bromophenol blue moved to about 1 cm from the bottom of the gel and stopped. Then, the proteins were electro-transferred onto polyvinylidene fluoride (PVDF) membranes (Merck Millipore, Darmstadt, Germany). The transfer buffer was prepared by dissolving 3.03 g Tris base, 14.42 g glycine and 150 mL methanol in double-distilled water to 1,000 mL. The transfer process was performed at a constant voltage of 100 V. Depending on protein molecular weight, Claudin-1 was transferred for 25 min, β-actin and Occludin were transferred for 55 min and Zona occludens-1 (ZO-1) was transferred for 2 h.

After the transfer, the membrane was blocked with 1 × Tris-buffered saline-Tween (TBST) buffer containing 5% fat-free milk (15 mmol/L Tris-HCl, 150 mmol/L NaCl, 0.1% Tween 20 and 5% fat-free milk; pH 7.4) at room temperature for 1 h, and then the membrane was incubated with the primary antibody overnight at 4 °C. The specific primary antibodies used included beta-actin (β-actin) (1:1,000; Cell Signaling Technology, Danvers, USA), ZO-1, Occludin, and Claudin-1(1:1,000; Proteintech, Chicago, USA). After overnight, the membrane was washed three times with 1 × TBST for 10 min each, and then incubated with antirabbit IgG HRP-conjugated secondary antibody (1:2,000; Cell Signaling Technology, Danvers, U.S.) at room temperature for 1 h. After secondary antibody incubation, the membrane was washed three times with 1 × TBST for 10 min each time. Finally, the target bands were visualized through an automatic chemiluminescence/fluorescence image analysis system (Tanon 5200 Multi, Shanghai, China). The gray value of band was measured by the ImageJ (version 1.8.0), and the expression of each target protein was expressed as the target protein/β-actin protein ratio.

### Statistical analysis

All data were subjected to one-way analysis of variance for a completely randomized design using the general linear model procedure of SPSS (version 20, IBM, Chicago, USA) and expressed as the means ± standard error of mean (SEM). Statistical differences among the treatments were separated by Tukey test. In addition, we normalized the data by subsampling to equalize the OTU sequence. Microbiota relative abundance differences were assessed using one-way analysis of variance with Tukey test for multiple comparisons. Statistical significance was set at *P* <  0.05, whereas *P* values between 0.05 and 0.10 were considered as a trend.

## Results

### Colonic antioxidant properties and morphology

As shown in Table 1, compared with the CON group, the LPS challenge significantly increased the MDA concentration and ROS activity in the colon mucosal tissue of suckling piglets (*P* <  0.05). In addition, it significantly reduced the activities of GSH-Px, SOD and T-AOC (*P* < 0.05). Compared with the LPS-CON group, the concentration of MDA and the activity of ROS in the colonic mucosa tissue of the LPS-GOS group were significantly reduced (*P* < 0.05), and the activity of SOD tended to increase (*P* = 0.068).

**Table 1:** The effects of GOS on antioxidant properties in the colonic mucosa of LPS-challenged suckling piglets

Items	CON	LPS-CON	LPS-GOS	SEM	*P*-value
MDA, nmol/mg protein	1.20^b^	1.73^a^	1.25^b^	0.09	0.014
ROS, U/mg protein	108.13^b^	145.97^a^	112.20^b^	6.34	0.017
GSH-PX, U/mg protein	21.60^a^	15.86^b^	17.04^ab^	0.91	0.013
SOD, U/mg protein	41.71^a^	28.41^b^	36.52^ab^	1.84	0.004
T-AOC, U/mg protein	5.97^a^	4.18^b^	5.21^a^	0.22	< 0.001

Values are expressed as mean ± *SEM*, *n* = 6. *CON* control group, *LPS-CON* piglets challenged with *LPS*, *LPS-GOS* piglets fed with *GOS* and challenged with *LPS*. *MDA* malondialdehyde, *ROS* reactive oxygen species, *GSH-Px* glutathione peroxidase, *SOD* superoxide dismutase, *T-AOC* total antioxidant capacity. *P* values < 0.05 were considered significant, *P* values between 0.05 and 0.10 were considered as a tendency. Different superscript lowercase letters on the same line indicate significant differences (*P* < 0.05)

**Fig. 1:**
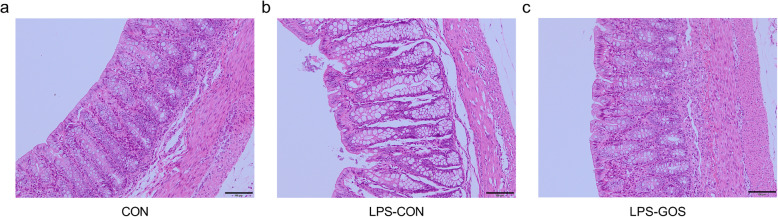
Effects of GOS on colonic epithelial morphology in LPS-challenged suckling piglets. **a** CON, control group. **b** LPS-CON, piglets challenged with LPS. **c** LPS-GOS, piglets fed with GOS and challenged with LPS

As shown in Fig. 1, compared with the CON group, LPS challenge resulted in an obvious injury and shedding of colonic mucosa (Fig. 1b). However, the colonic mucosa of piglets in the LPS-GOS group was only slightly injured and shed (Fig. 1c). There were no significant differences in colonic crypt depth and mucosal thickness among the three groups (Table 2).

**Table 2:** The effects of GOS on the colonic morphology of LPS-challenged suckling piglets

Items	CON	LPS-CON	LPS-GOS	SEM	*P*-value
Crypt depth, μm	265.85	292.14	275.53	6.74	0.297
Mucosal thickness, μm	32.86	91.62	76.30	16.49	0.529

Values are expressed as mean ± *SEM*, *n* = 6. *CON* control group, *LPS-CON* piglets challenged with *LPS*, *LPS-GOS* piglets fed with *GOS* and challenged with *LPS*

### Diversity of the colonic mucosa-associated microbiota

**Fig. 2:**
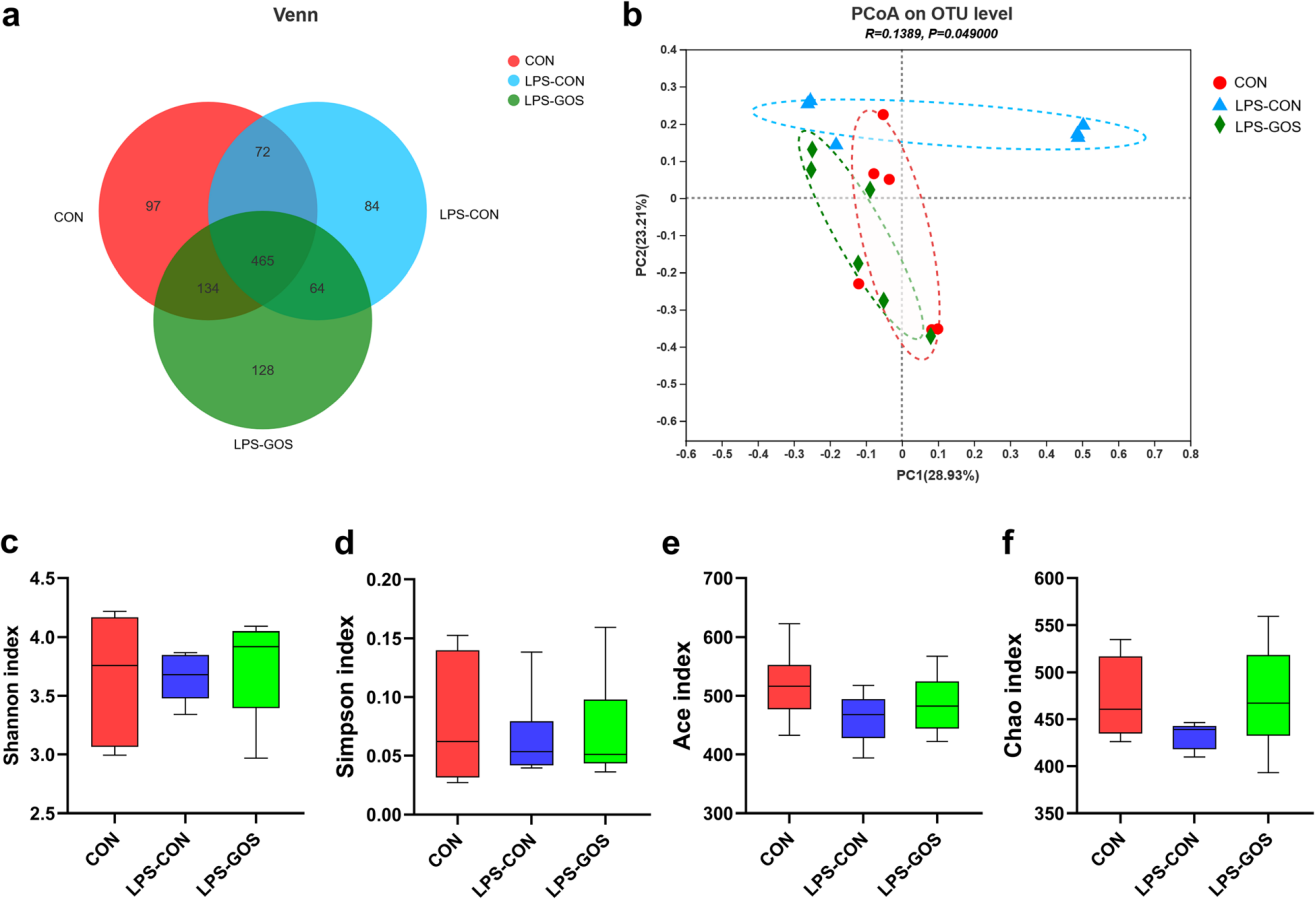
Effects of GOS on the diversity of colonic mucosa microbiota in LPS-challenged suckling piglets. **a** Venn diagram of core operational taxonomic units in the colonic mucosa. **b** Principal coordinate analysis (PCoA) on colonic microbiota, ANOSIM analysis was used to assess significant differences. **c** the Shannon index of colonic mucosa microbiota. **d** the Simpson index of colonic mucosa microbiota. **e** the Ace index of colonic mucosa microbiota. **f** the Chao index of colonic mucosa microbiota. Values are expressed as mean ± SEM, *n* = 6. CON, control group; LPS-CON, piglets challenged with LPS; LPS-GOS, piglets fed with GOS and challenged with LPS

In this study, we obtained a total of 713,963 sequences in the V3–V4 region, with an average of 39,665 sequences per sample. The OTU was clustered with 97% similarity. There were 768, 685 and 791 core OTUs in the CON group, LPS-CON group and LPS-GOS group, respectively, and 465 core OTUs were common in the three groups (Fig. 2a). The effects of three treatments on colonic mucosa-associated microbiota alpha and beta diversity were investigated. As shown in Fig. 2c–f, the α diversity index including Shannon, Simpson, Ace and Chao indexes were not significantly different among the three groups. β diversity, PCoA analysis based on bray−curtis distance, showed significant separations of colonic mucosal microbial composition among the three groups (Fig. 2b).

### Bacterial abundance in the colonic mucosa

**Fig. 3:**
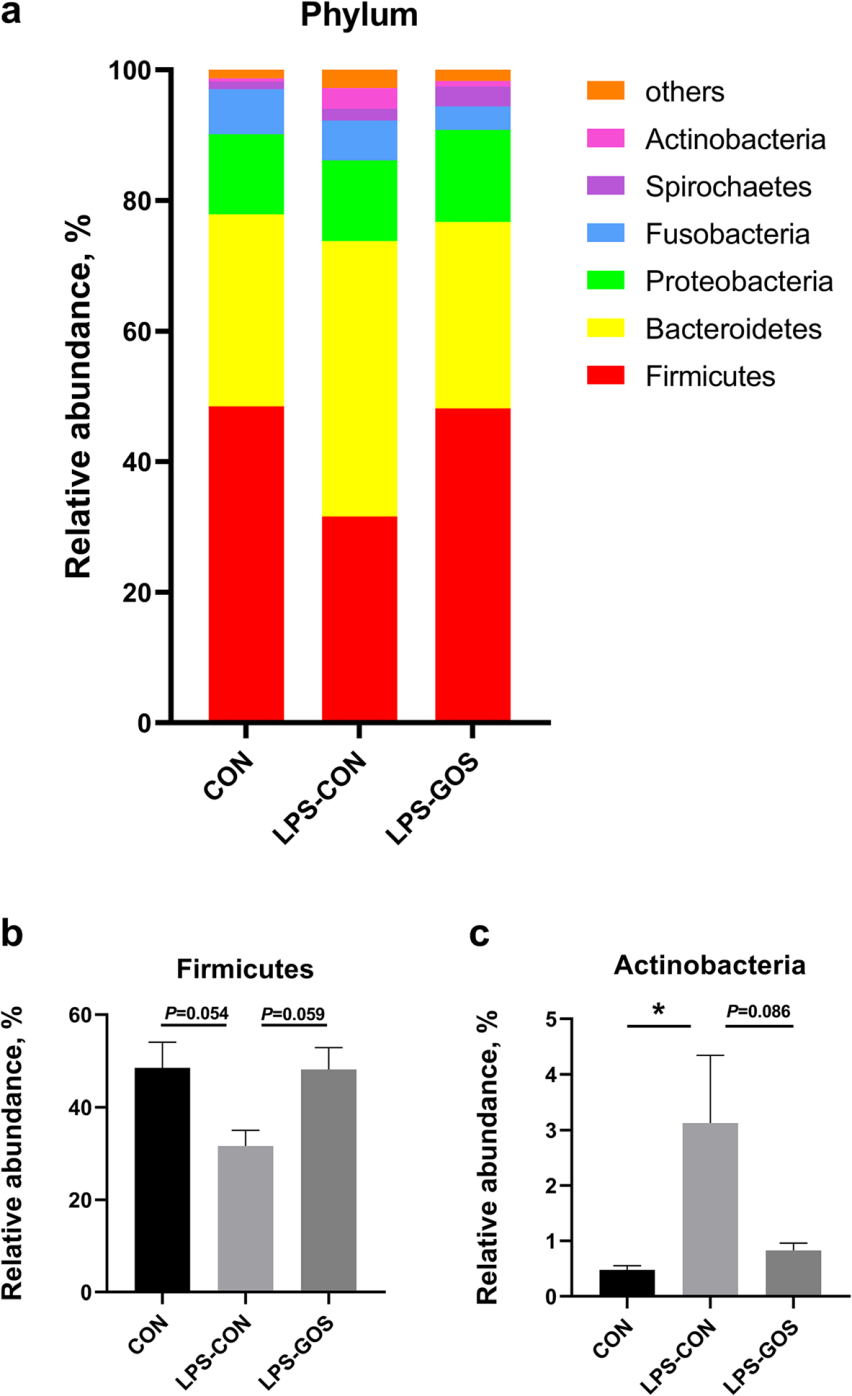
Effects of GOS on the relative abundance of colonic mucosal microbiota at the phylum level. **a** Phylum level composition. **b** The change of Firmicutes. **c** The change of Actinobacteria. *P* values < 0.05 were considered significant. Values are expressed as mean ± SEM, *n* = 6. CON, control group; LPS-CON, piglets challenged with LPS; LPS-GOS, piglets fed with GOS and challenged with LPS. * means *P* < 0.05, ** means *P* < 0.01

The colonic mucosa microbiota composition in the three treatment groups is shown in Fig. 3. At the phylum level, Firmicutes, Bacteroidetes, and Proteobacteria are the mainly dominant phyla (Fig. 3a). Statistical analysis showed that the relative abundance of Firmicutes in the LPS-CON group tended to decrease (*P* = 0.054) while the relative abundance of Actinobacteria increased significantly (*P* < 0.05) compared with the CON group. However, the relative abundance of Firmicutes in the LPS-GOS group tended to increase (*P* = 0.059), and the relative abundance of Actinobacteria tended to decrease (*P* = 0.086) compared with the LPS-CON group (Fig. 3b-c).

**Fig. 4:**
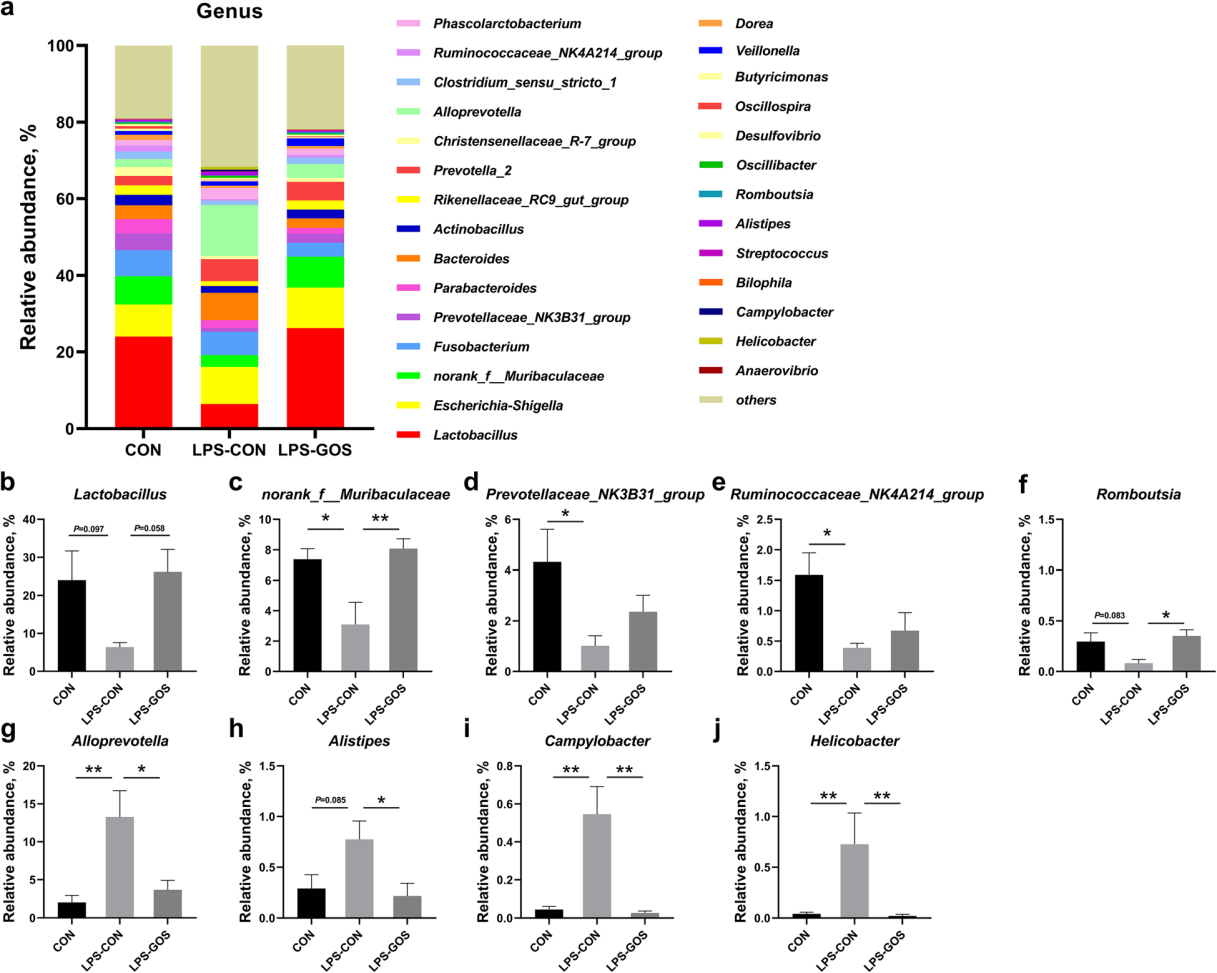
Effects of GOS on the relative abundance of colonic mucosal microbiota at the genus level. **a** Genus level composition. **b** The change of *Lactobacillus.* **c** The change of *norank_f__Muribaculaceae.* **d** The change of *Prevotellaceae_NK3B31_group*. **e** The change of *Ruminococcaceae_NK4A214_group.* **f** The change of *Romboutsia*. **g** The change of *Alloprevotella*. **h** The change of *Alistipes*. **i** The change of *Campylobacter*. **j** The change of *Helicobacter*. *P* values < 0.05 were considered significant, *P* values between 0.05 and 0.10 were considered as a tendency. Values are expressed as mean ± SEM, *n* = 6. CON, control group; LPS-CON, piglets challenged with LPS; LPS-GOS, piglets fed with GOS and challenged with LPS. * means *P* < 0.05, ** means *P* < 0.01

At the genus level, genera with relative abundance greater than 0.1% in at least one treatment group are shown in Fig. 4a. According to the statistical analysis, LPS challenge significantly reduced the relative abundance of *norank_f__Muribaculaceae, Prevotellaceae_NK3B31_group,* and *Ruminococcaceae_NK4A214_group* (*P* < 0.05), tended to reduce the relative abundance of *Lactobacillus* (*P* = 0.097) and *Romboutsia* (*P* = 0.083) (Fig. 4b–f) compared with the CON group. In addition, the relative abundance of *Alloprevotella*, *Campylobacter* and *Helicobacter* (*P* < 0.01) was significantly increased and the relative abundance of *Alistipes* (*P* = 0.085) tended to increase (Fig. 4g–j) in the LPS-CON group. On the other hand, the relative abundance of *norank_f__Muribaculaceae* (*P* < 0.01) and *Romboutsia* (*P* < 0.05) in the LPS-GOS group was increased significantly, and the relative abundance of *Lactobacillus* (*P* = 0.058) tended to increase (Fig. 4b, c and f) compared with the LPS-CON group. In addition, the relative abundance of *Alloprevotella*, *Alistipes* (*P* < 0.05), *Campylobacter* and *Helicobacter* (*P* < 0.01) was decreased significantly (Fig. 4g–j).

### The pH value and microbial metabolites in colonic digesta

The effects of GOS on pH value and microbial metabolites of colonic digesta in suckling piglets challenged with LPS are shown in Table 3. Compared with the CON group, LPS challenge significantly increased the pH value (*P* < 0.05) of colonic digesta, significantly reduced the concentrations of acetate, butyrate (*P* < 0.05) and total SCFAs (*P* < 0.05) in colonic digesta, and tended to reduce the concentration of lactate (*P* = 0.051). However, compared with the LPS-CON group, the pH of colonic digesta was significantly decreased (*P* < 0.05) and the concentrations of acetate, butyrate, total SCFAs and lactate in the colonic digesta were significantly increased (*P* < 0.05) in the LPS-GOS group.

**Table 3:** The effects of GOS on colonic pH value, SCFAs and lactic acid concentrations in colonic digesta of LPS-challenged suckling piglets

Items	CON	LPS-CON	LPS-GOS	SEM	*P*-value
pH value	6.45 ^b^	6.72 ^a^	6.47 ^b^	0.05	0.020
Acetate, μmol/g digesta	25.14 ^a^	20.15 ^b^	24.47 ^a^	0.83	0.018
Propionate, μmol/g digesta	12.60	10.06	10.99	0.56	0.181
Isobutyrate, μmol/g digesta	1.19	1.10	1.16	0.03	0.533
Butyrate, μmol/g digesta	6.65 ^a^	4.62 ^b^	6.72 ^a^	0.34	0.007
Isovalerate, μmol/g digesta	2.10	1.90	1.97	0.12	0.804
Valerate, μmol/g digesta	2.01	1.10	1.36	0.18	0.091
Total SCFAs, μmol/g digesta	49.69 ^a^	38.93 ^b^	46.68 ^a^	1.57	0.006
Lactate, μmol/g digesta	1.58 ^ab^	1.26 ^b^	1.62 ^a^	0.06	0.021

Values are expressed as mean ± *SEM*, *n* = 6. *CON* control group, *LPS-CON* piglets challenged with *LPS*, *LPS-GOS* piglets fed with *GOS* and challenged with *LPS*. *P* values < 0.05 were considered significant, *P* values between 0.05 and 0.10 were considered as a tendency. Different superscript lowercase letters on the same line indicate significant differences (*P* < 0.05)

### The mRNA expression of mucins in the colonic mucosa

**Fig. 5:**
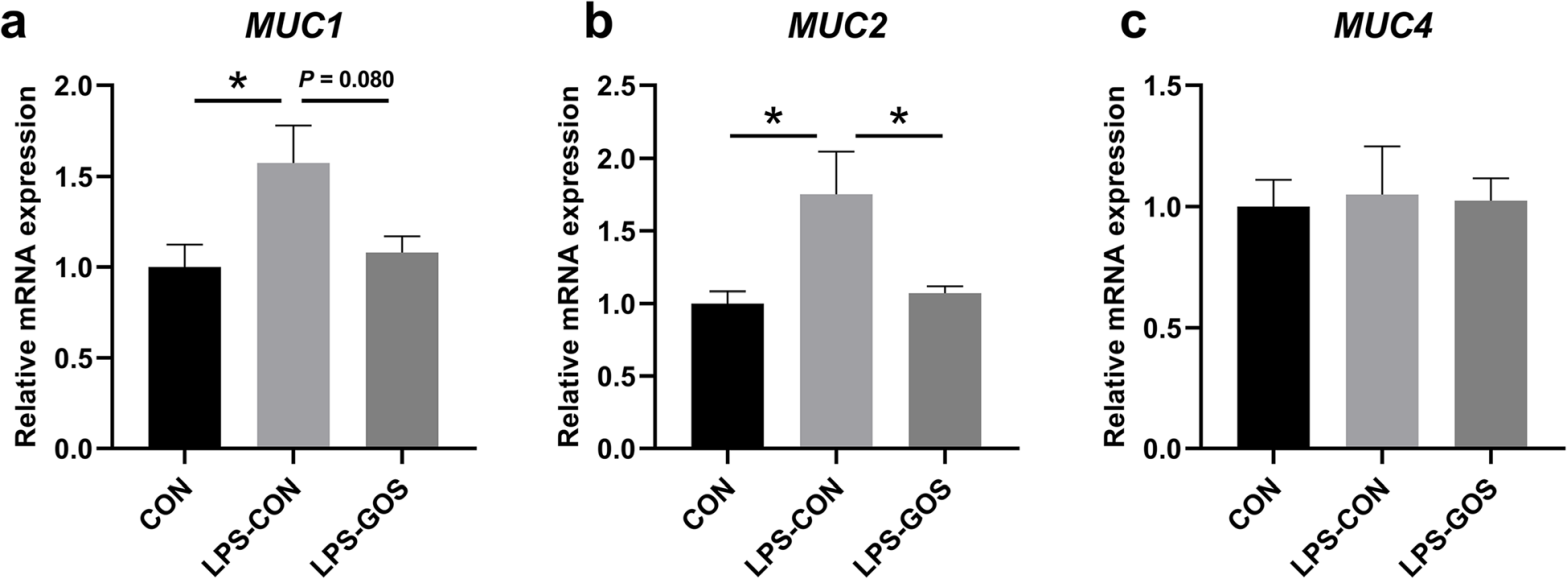
Effects of GOS on the relative mRNA expression of mucin in suckling piglets. The values were calculated relative to the expression of GAPDH with formula 2-ΔΔCt. **a** The change of MUC1. **b** The change of MUC2. **c** The change of MUC4. *P* values < 0.05 were considered significant, *P* values between 0.05 and 0.10 were considered as a tendency. Values are expressed as mean ± SEM, *n* = 6. CON, control group; LPS-CON, piglets challenged with LPS; LPS-GOS, piglets fed with GOS and challenged with LPS. * means *P* < 0.05, ** means *P* < 0.01

As shown in Fig. 5, the relative mRNA expression of Mucin 1 (*MUC1*) and Mucin 2 (*MUC2*) in colonic mucosa of piglets in the LPS-CON group was significantly higher than that in the CON group (*P* < 0.05). Compared with the LPS-CON group, the relative mRNA expression of *MUC1* in the LPS-GOS group tended to decrease (*P* = 0.080), and the relative mRNA expression of *MUC2* significantly decreased (*P* < 0.05). There was no significant change in *MUC4* expression among the three groups.

### The protein expression level of tight junction protein in colonic mucosa

**Fig. 6:**
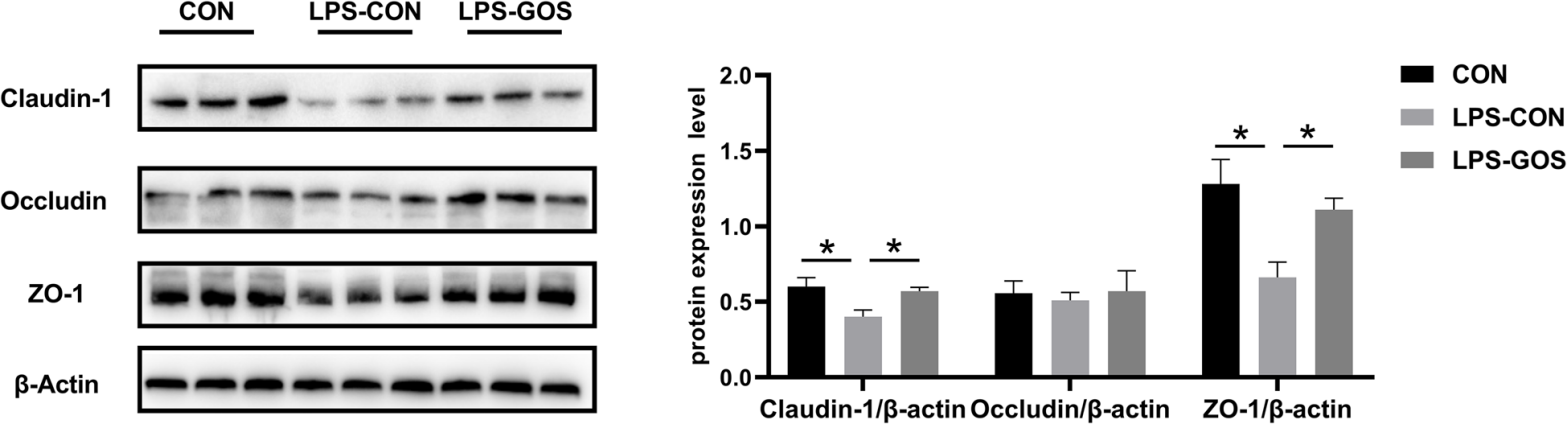
Effects of GOS on the protein expression of barrier proteins in suckling piglets. *P* values < 0.05 were considered significant. Values are expressed as mean ± SEM, *n* =  6. CON, control group; LPS-CON, piglets challenged with LPS; LPS-GOS, piglets fed with GOS and challenged with LPS. * means *P* < 0.05, ** means *P* < 0.01

As shown in Fig. 6, the protein expression of Claudin-1 and ZO-1 in the colonic mucosa were significantly decreased in the LPS-CON group compared with those in the CON group (*P* < 0.05). In addition, the protein expression of Claudin-1 and ZO-1 was significantly increased in the LPS-GOS group compared with those in the LPS-CON group (*P* < 0.05). The protein expression of Occludin was not significantly different among the three groups.

### The concentration of cytokines in the colonic mucosa

**Fig. 7:**
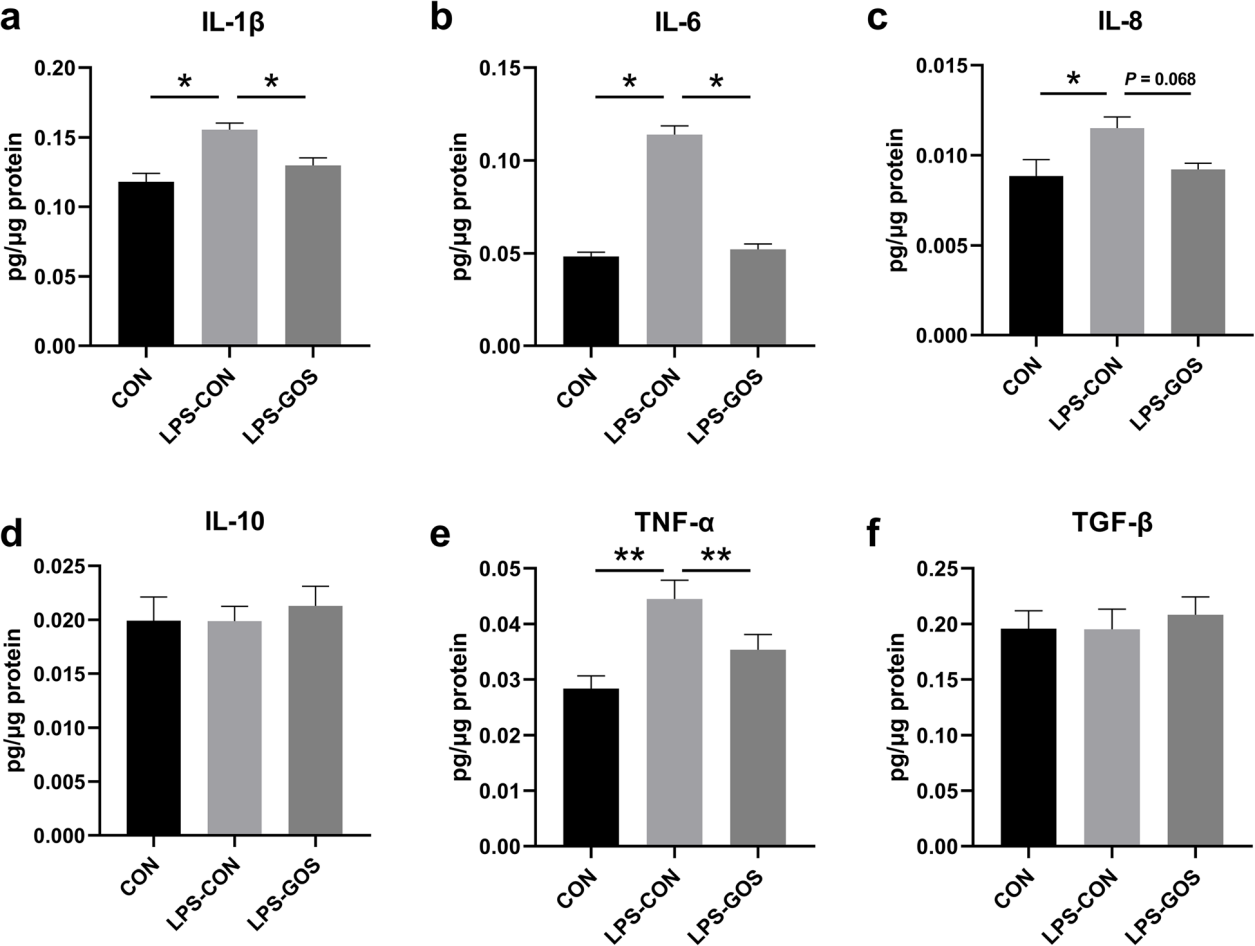
Effects of GOS on the cytokine concentration in suckling piglets. **a** The change of IL-1β. **b** The change of IL-6. **c** The change of IL-8. **d** The change of IL-10. **e** The change of TNF-α. **f** The change of TGF-β. *P* values < 0.05 were considered significant, *P* values between 0.05 and 0.10 were considered as a tendency. Values are expressed as mean ± SEM, *n* =  6. CON, control group; LPS-CON, piglets challenged with LPS; LPS-GOS, piglets fed with GOS and challenged with LPS. * means *P* < 0.05, ** means *P* < 0.01

As shown in Fig. 7a–c and Fig. 7e, LPS challenge significantly increased the concentrations of IL-1β, IL-6, IL-8 (*P* < 0.05) and TNF-α (*P* < 0.01) in colonic mucosa compared with the CON group. The concentrations of IL-1β, IL-6 (*P* < 0.05) and TNF-α (*P* < 0.01) in the LPS-GOS group were decreased significantly, and the concentration of IL-8 (*P* = 0.068) tended to decrease compared with those in the LPS-CON group. No significant difference was observed in the concentration of IL-10 and TGF-β among the three groups (Fig. 7d–f).

### The concentration of CD14 in colonic mucosa and the relative mRNA expression of key molecules in NF-κB signaling pathway

**Fig. 8:**
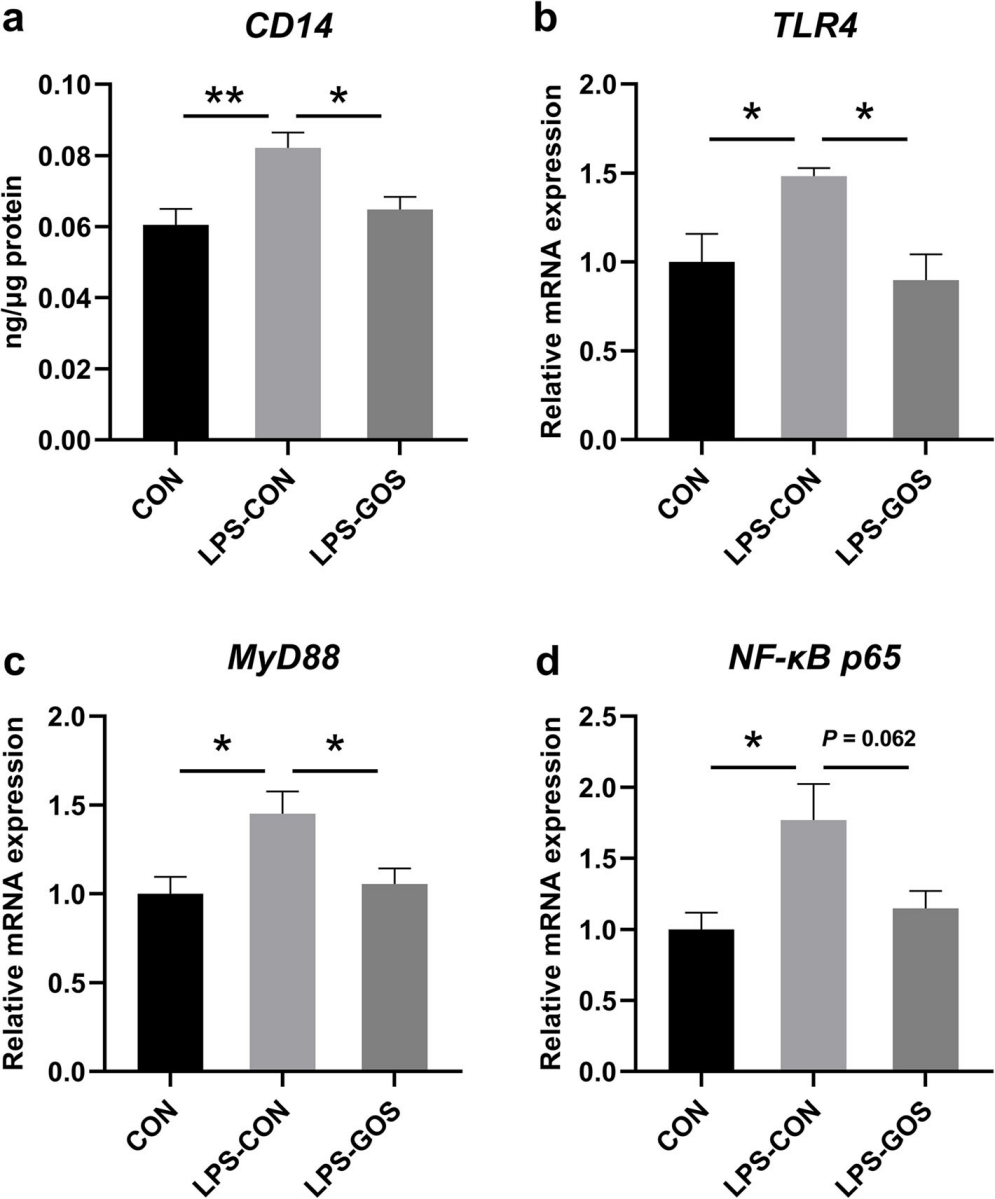
Effects of GOS on inflammatory pathway related factors in suckling piglets. **a** The change of CD14. **b** The change of TLR4. **c** The change of MyD88. **d** The change of NF-κB p65. *P* values < 0.05 were considered significant, *P* values between 0.05 and 0.10 were considered as a tendency. Values are expressed as mean ± SEM, *n* = 6. CON, control group; LPS-CON, piglets challenged with LPS; LPS-GOS, piglets fed with GOS and challenged with LPS. * means *P* < 0.05, ** means *P* < 0.01

As shown in Fig. 8 (a–d), LPS challenge significantly increased the concentration of CD14 (*P* < 0.01) and the relative mRNA expression of *TLR4, MyD88* and *NF-κB p65* (*P* < 0.05) in the colonic mucosa compared with the CON group. The concentration of CD14 in the colonic mucosa was significantly decreased and the relative mRNA expressions of *TLR4* and *MyD88* in the colonic mucosa were significantly decreased in the LPS-GOS group while the relative mRNA expression of *NF-κB p65* tended to decrease (*P* = 0.062) compared with those in the LPS-CON group.

## Discussion

The first week after birth is a critical period for the colonization of the intestinal microbiota in piglets, and the colonization and succession of the intestinal microbiota in early life is the key factor that affects the establishment of specific microbiota composition and phenotype of newborns [20, 21]. In addition, the development of innate and adaptive immune responses, as well as the homeostasis of intestinal barrier function are largely influenced by intestinal microbial colonization [22]. As we know, the colon is the intestinal segment with the largest number of microorganisms and vigorous microbial metabolic activity [23]. It has been pointed out that the intestinal microbiota has the most significant modulatory effect on intestinal immunity of piglets in the colon [24]. Intraperitoneal injection of LPS is widely used to construct an animal model of acute intestinal inflammatory injury [8]. Thus, to further highlight the beneficial effects of GOS, we constructed a piglet colonic inflammation model with intraperitoneal LPS, focusing on the effects of GOS on the colonization of colonic microbiota, colonic inflammatory response and barrier function in LPS-challenged suckling piglets. This could provide a good theoretical support for the application of GOS in feed additives.

In this experiment, we observed that LPS caused the injury of the colon morphology of piglets, while GOS alleviated the injury to the colon morphology and maintained the integrity of the colon. Normally, intestinal oxidative stress causes the injury of intestinal morphology, and it has been reported that LPS would cause intestinal oxidative stress in piglets, injure intestinal tissue and disrupt the integrity of intestinal morphology [25, 26]. In the results, we found that LPS challenge significantly increased the concentration of MDA and the activity of ROS, and decreased the activity of T-AOC in the colonic mucosa. ROS are reactive oxygen free radicals generated from cellular metabolism in the animal body, and the accumulation of ROS is a key factor causing intestinal oxidative stress [27]. In addition, ROS will attack lipids in the body, causing them to undergo lipid peroxidation and ultimately produce MDA. MDA is cytotoxic and is also an indicator of oxidative stress status [28]. While T-AOC is commonly used to assess the total antioxidant capacity of tissues, reflecting the balance status between antioxidant and oxidative systems. Thus, the increased levels of MDA and ROS, and the decreased activity of T-AOC indicate that LPS cause oxidative stress in the colon. In addition, the inhibition of the activities of important antioxidant enzymes SOD and GSH-Px in the intestine by LPS further aggravated the oxidative stress of the colon. On the other hand, we found that GOS reduced the levels of MDA and ROS in the colonic mucosa, and increased the levels of SOD and T-AOC of LPS-challenged suckling piglets. It shows that GOS alleviate the oxidative stress of the colon caused by LPS to a certain extent. Therefore, these results suggest the fact that GOS intervention is beneficial to maintain the integrity of the colon morphology of LPS-challenged suckling piglets.

In the present study, we observed that Firmicutes and Bacteroidetes were the most abundant phyla in the colonic mucosa of suckling piglets, which was consistent with the study of Li et al. [29]. The relative abundance of Firmicutes was decreased and Actinobacteria was increased in the LPS-CON group compared with that in the CON group. However, the relative abundance of Firmicutes was increased and Actinobacteria was decreased in the LPS-GOS group compared with that in the LPS-CON group. The increased relative abundance of Firmicutes is beneficial to intestinal health, as it contains a large number of SCFA-producing bacteria, such as butyrate-producer *Eubacterium rectale and Eubacterium Hallii*, which help to maintain intestinal health [30]. Actinobacteria is reported to be part of the symbiosis intestinal microbiota, but a high proportion of Actinobacteria is associated with intestinal inflammatory diseases and colon cancer [31]. Therefore, our results suggested that GOS could increase the relative abundance of Firmicutes and decrease the relative abundance of Actinobacteria in the colonic mucosa of LPS-challenged piglets.

At the genus level, we found that LPS stimulation resulted in the changes of the relative abundance of nine genera, and the changes in seven genera were reversed by GOS intervention. LPS challenge decreased the relative abundance of *Lactobacillus* and *norank_f__Muribulaceae* and increased the relative abundance of *Alloprevotella*, while GOS intervention prevented the LPS-induced reduction of the relative abundance of *Lactobacillus* and *norank_f__Muribulaceae* and the increase of *Alloprevotella*. *Lactobacillus* is a common probiotic that has been shown to colonize in the intestine and become a stable member of the entire intestinal microbial community shortly after birth in piglets [32]. Miyauchi et al. have reported that *Lactobacillus* could maintain the colonic epithelial barrier function and alleviate the colonic inflammatory response in dextran sulfate sodium (DSS)-induced colitis mice [33]. In addition, *Lactobacillus* would prevent intestinal pathogen infection and protect intestinal health by producing metabolites such as lactic acid and competing for mucosal binding sites with gram-negative pathogens such as *Campylobacter* [34, 35]. Our results suggest that the increased relative abundance of *Lactobacillus* may contribute to the protective effect of GOS on colon health in LPS-challenged suckling piglets. The function of *norank_f__Muribaculaceae* is largely unknown, but Liu et al. found that the relative abundance of *norank_f__Muribaculaceae* was positively correlated with the metabolites enriched in the feces of healthy mice [36]. This suggested that *norank_f__Muribaculaceae* might be a bacterium enriched in the healthy intestinal environment. The relative abundance of *Alloprevotella* increased in the colon mucosa of ulcerative colitis carcinogenesis mice [37]. Additionally, our results are consistent with Wang’s finding that the early GOS intervention can reduce the relative abundance of *Alloprevotella* in the colonic mucosa of suckling piglets [16]. Hence, we speculated that the significant decrease in the relative abundance of *Alloprevotella* in the LPS-GOS group compared with that in the LPS-CON group might be an indication of GOS alleviating the colonic inflammation caused by LPS in piglets. Notably, LPS stimulation led to an increase in the relative abundance of potential pathogenic bacteria *Alistipes*, *Campylobacter* and *Helicobacter*, but GOS could significantly reduce their relative abundance. *Alistipes*, *Campylobacter* and *Helicobacter* can cause inflammatory diseases in the intestine, resulting in gastrointestinal dysfunction [38–40]. Therefore, in general, our results suggested that GOS contribute to maintaining colonic mucosal microbiota homeostasis in LPS-challenged suckling piglets, promoting the colonization of beneficial bacteria and reducing the colonization of potentially pathogenic bacteria.

SCFAs are the main metabolites of intestinal microbiota. Through the production of metabolites including SCFAs, lactic acid and bile acid, the intestinal microbiota established a close association with various physiological and immune responses of the host [41]. Here, we observed significant changes in the concentrations of acetate, butyrate and total SCFAs in the colonic lumen. LPS significantly reduced the concentrations of acetate, butyrate, and total SCFAs. This may be due to the fact that LPS reduced the relative abundance of Firmicutes, *Romboutsia*, *Prevotellaceae_NK3B31_group* and *Ruminococcaceae_NK4A214_group*, which produce SCFAs including acetate and butyrate by fermenting dietary components [30, 42–44]. GOS increased the concentration of SCFAs by significantly increasing the relative abundance of *Firmicutes* and *Romboutsia* in LPS-challenged suckling piglets. SCFAs are key factors modulating the intestinal epithelial barrier function and intestinal immunity of the host, and have a variety of beneficial effects on the host, including maintaining the intestinal barrier function and anti-inflammatory effects [45]. Meanwhile, the homeostasis of the colonic mucosa is largely influenced by the concentration of SCFAs which are produced by colonic microbiota. Butyrate, which is a major nutrient involved in the repair and regeneration of colonic epithelial cells, plays an important role in the health of the colonic mucosa [46, 47]. In addition, we found that LPS decreased the concentration of lactate in the colonic lumen, while GOS significantly increased the concentration of lactate, which was consistent with the change trend of *Lactobacillus*. Neal-McKinney et al. reported that lactate is an important factor for reducing *Campylobacter* growth in livestock [48]. Our finding of the decreased relative abundance of *Campylobacter* in the colonic mucosa was consistent with this idea. Notably, lactate can be further metabolized to SCFAs, especially butyrate, by lactate-utilizing bacteria such as *Megasphaera* in the intestine [49, 50]. With the increase of SCFAs and lactate concentration, the pH value of colon in the LPS-GOS group decreased significantly compared with that in the LPS-CON group. A lower pH value is beneficial to maintain the acidic environment in the colon, thereby inhibiting the growth and colonization of pathogenic bacteria such as *E. coli* and *Salmonella* [51]. Therefore, the increased concentrations of SCFAs and lactate in the LPS-GOS group piglets contribute to the colonic mucosal homeostasis.

As mentioned above, SCFAs have been shown to have anti-inflammatory effects in the intestine. In order to further explore the inflammatory status of the piglet colon, we determined the levels of cytokines. We found that LPS stimulation significantly increased the concentrations of pro-inflammatory cytokines IL-1β, IL-6, IL-8 and TNF-α in the colonic mucosa, without significant effect on the anti-inflammatory cytokines IL-10 and TGF-β, which indicated that LPS promote the secretion of proinflammatory cytokines to induce inflammatory response. However, GOS significantly inhibited the increase of IL-1β, IL-6, IL-8 and TNF-α concentration caused by LPS. Studies have shown that LPS induce the release of a series of pro-inflammatory cytokines through binding to TLR4 and stimulating the downstream signaling molecule MyD88 as well as NF-κB p65 [52]. Zanoni et al. found that the activation of TLR4/NF-κB pathway caused by bacterial endotoxin in the traditional sense was modulated by CD14 because of the high affinity between CD14 and LPS. CD14 is the first pattern recognition receptor that binds to LPS, can transmit LPS to TLR4 and control TLR4 entry into the endosomal network through endocytosis [53]. In this study, we found that the decreased concentrations of pro-inflammatory cytokines IL-1β, IL-6, IL-8 and TNF-α in the LPS-GOS group were consistent with the decreased concentration of CD14 and the relative mRNA expression of *TLR4, MyD88* and *NF-κB p65* in the colonic mucosa. The NF-κB signaling pathway plays a key modulatory role in the inflammatory response, and Meijer et al. have found that SCFAs can inhibit the activation of NF-κB and contribute to the reduced secretion of pro-inflammatory cytokines [54]. Thus, in our study, GOS supplement increased the relative abundance of SCFAs producing bacteria, accompanied by an increase in a higher concentration of SCFAs, which may contribute to the reduced secretion of pro-inflammatory cytokines in LPS-challenged suckling piglets.

Altered intestinal microbiota and SCFAs have important effects on the maintenance of intestinal barrier function [55]. In addition, intestinal inflammation is closely associated with the intestinal barrier. IL-1β, IL-6 and IL-8 were reported to impair intestinal barrier function by rearranging tight junction proteins, while TNF-α significantly disrupts the tight junctions between intestinal epithelial cells [56]. Hence, GOS alleviated the colonic inflammation caused by LPS challenge in piglets, which contribute to the maintenance of colonic mucosal barrier function. Intestinal tight junctions (TJs) mainly include Claudin, Occludin and zonula occludens (ZO) families, which are the main components of the physical barrier of intestinal epithelium and determine the selective permeability of intestinal epithelium [57]. TJs are vulnerable to the exposure of external stressors, which cause unregulated transport and diffusion of macromolecules such as endotoxin and antigen into mucosa, leading to local or systemic inflammation [58]. This explains why LPS stimulation significantly reduced the protein expression of Claudin-1 and ZO-1 in present study. Some studies have reported that probiotics such as *Lactobacillus plantarum* can increase the expression of TJs in intestinal epithelium of piglets, and SCFAs could enhance intestinal barrier function by activating AMPK signaling pathway [59, 60]. In our study, the protein expression of Claudin-1 and ZO-1 in the LPS-GOS group was significantly higher than that in the LPS-CON group. Mucins play a key role in the chemical barrier formed by the epithelial mucus layer, and the number and maturity of mucins covering the intestinal epithelial surface are important factors influencing the optimal disease resistance [61]. Interestingly, we found that the relative mRNA expression of *MUC1* and *MUC2* in the LPS-CON group was significantly higher than that in the CON group. Studies have pointed out that LPS stimulation can cause mucosal mucin secretion to be excessive, and this change is usually accompanied by bacterial inflammation of the mucosa [62]. Therefore, we speculated that the significant increase of the relative mRNA expression of *MUC1* and *MUC2* were associated with LPS which stimulate piglets to produce an acute inflammation response 2 h after intraperitoneal injection, and resulted in a large amount of mucus secreted by the colon in this study. The relative mRNA expression of *MUC*1 and *MUC2* in the LPS-GOS group was decreased compared with that in the LPS-CON group, probably because GOS help to alleviate the inflammation response caused by LPS. Therefore, GOS help to maintain the colonic epithelial barrier function in LPS-challenged suckling piglets, which may be mainly related to the changes of colonic mucosa-associated microbiota composition and metabolites, as well as the alleviation of inflammation.

## Conclusion

In summary, this study demonstrated that a daily dose of GOS supplement for the first 13 days of life could modulate the colonic mucosal microbiota in LPS-challenged suckling piglets, which was beneficial to the construction of a healthy colonic environment. In addition, GOS increased the concentration of SCFAs in the colonic lumen, and contribute to alleviating LPS-induced colonic inflammatory response and ultimately help to maintain the colonic barrier function.

## Supplementary Information

**Additional file 1: Table S1.** Primer sequences for quantitative real-time PCR analysis.

## Data Availability

All data generated or analyzed during this study are available from the corresponding author upon reasonable request.

## Abbreviations

BCA: Bicinchoninic acid
GAPDH: Glyceraldehyde-3-phosphate dehydrogenase
CD14: Cluster of differentiation 14
GOS: Galacto-oligosaccharides
GSH-Px: Glutathione peroxidase
IL-1β: Interleukin 1β
IL-6: Interleukin 6
IL-8: Interleukin 8
IL-10: Interleukin 10
LPS: Lipopolysaccharides
MDA: Malondialdehyde
MUC1: Mucin 1
MUC2: Mucin 2
MUC4: Mucin 4
MyD88: Myeloid differentiation primary response 88
NF-κB p65: Nuclear factor kappa B p65
ROS: Reactive oxygen species
SCFAs: Short chain fatty acids
SOD: Superoxide dismutase
T-AOC: Total antioxidant capacity
TGF-β: Transforming growth factor-β
TJs: Tight junctions
TLR4: Toll-like receptor 4
TNF-α: Tumor necrosis factor-α
ZO-1: Zonula occluden 1
