# Supplementary material for: Galacto-oligosaccharides improve barrier function and relieve colonic inflammation via modulating mucosa-associated microbiota composition in lipopolysaccharides-challenged piglets
Source: J Anim Sci Biotechnol. 2021 Aug 11;12:92. doi: 10.1186/s40104-021-00612-z (PMC8356462; doi:10.1186/s40104-021-00612-z)
Supplement: Supplementary file 1 — Additional file 1: Table S1. Primer sequences for quantitative real-time PCR analysis. [file 40104_2021_612_MOESM1_ESM.docx]

**Table S1.** Primer sequences used for Real-time quantitative PCR

| Genes | Forward primer (5'-3') | Reverse primer (5'-3') | Accession number |
| --- | --- | --- | --- |
| *MUC1* | GTGCCGCTGCCCACAACCTG | AGCCGGGTACCCCAGACCCA | XM_021089730.1 |
| *MUC2* | GGTCATGCTGGAGCTGGACAG | TGCCTCCTCGGGGTCGTCAC | XM_021082584.1 |
| *MUC4* | GATGCCCTGGCCACAGAA | TGATTCAAGGTAGCATTCATTTGC | XM_021068274.1 |
| *TLR4* | TCAGTTCTCACCTTCCTCCTG | GTTCATTCCTCACCCAGTCTTC | NM_001293316.1 |
| *MyD88* | CCCCAGCGATACCCAGTTTGT | ATCCGACGGCACCTCTTTTCA | NM_001099923.1 |
| *NF-κB p65* | AACCCCTTCCAAGTTCCCA | TCCCCGAGTTCCGATTCAC | NM_001114281.1 |
| *GAPDH* | ATGGTGAAGGTCGGAGTGAAC | ATGGTGAAGGTCGGAGTGAAC | NM_001206359.1 |

*MUC1*, Mucin 1; *MUC2*, Mucin 2; *MUC4*, Mucin 4; *TLR4*, Toll-like receptor 4; *MyD88*, myeloid differentiation primary response 88; *NF-κB-p65*, nuclear factor kappa B-p65; *GAPDH*, glyceraldehyde phosphate dehydrogenase.
